# Iatrogenic Ocular Surface Complications After Surgery for Ocular and Adnexal Tumors

**DOI:** 10.3390/cancers17091384

**Published:** 2025-04-22

**Authors:** Maria Angela Romeo, Andrea Taloni, Massimiliano Borselli, Alessandra Di Maria, Alessandra Mancini, Vincenzo Mollace, Giovanna Carnovale-Scalzo, Vincenzo Scorcia, Giuseppe Giannaccare

**Affiliations:** 1 Department of Ophthalmology, University Magna Graecia of Catanzaro, 88100 Catanzaro, Italy; oculistica.romeo.ma@gmail.com (M.A.R.); vscorcia@unicz.it (V.S.); 2 Department of Translational Medicine, University of Ferrara, 44121 Ferrara, Italy; 3 Department of Ophthalmology, Ospedali Privati Forlì “Villa Igea”, 47122 Forlì, Italy; 4 Istituto Internazionale per la Ricerca e Formazione in Oftalmologia, 47122 Forlì, Italy; 5 Department of Ophthalmology, IRCCS Humanitas Research Hospital, Rozzano, 20089 Milan, Italy; alessandra.di_maria@humanitas.it; 6 Department of Biomedical Sciences, Humanitas University, Pieve Emanuele, 20072 Milan, Italy; 7 Departemnt of Health Sciences, Institute of Research for Food Safety and Health (IRC-FSH), University Magna Graecia of Catanzaro, 88100 Catanzaro, Italy; 8 Eye Clinic, Department of Surgical Sciences, University of Cagliari, 09124 Cagliari, Italy

**Keywords:** iatrogenic ocular surface diseases, ocular tumor surgery, eyelid tumors, conjunctival tumors, uveal melanoma, eyelid malposition, symblepharon, epiphora, keratopathy, dry eye

## Abstract

**Background/Objectives:** The management of ocular tumors often necessitates surgery, either alone or in combination with radiotherapy, chemotherapy, or other modalities. While crucial for tumor control, these treatments can significantly impact the ocular surface, leading to both acute and chronic complications. This review examines iatrogenic ocular surface diseases resulting from oncologic interventions, emphasizing their pathophysiology, diagnostic challenges, and management strategies. **Methods:** A literature review was conducted to identify studies on iatrogenic ocular surface complications associated with ocular tumor treatments. **Results:** Ocular surface complications include direct damage from surgical manipulation, leading to corneal opacities and persistent epithelial defects, as well as dry eye disease secondary to postoperative chemosis. These disruptions may progress to more severe conditions such as keratopathy, corneal ulcers, limbal stem cell deficiency, and stromal scarring, further impairing visual function. Structural alterations contribute to eyelid malpositions—including ectropion, entropion, round eye, and lagophthalmos—which exacerbate exposure-related damage and ocular surface instability. In cases of uveal melanomas, the exposure of episcleral brachytherapy plaques can induce chronic conjunctival irritation, promoting adhesion formation and symblepharon. Surgical interventions disrupt ocular surface homeostasis, while radiotherapy and chemotherapy exacerbate these effects through cytotoxic and inflammatory mechanisms. **Conclusions:** Preventing and managing iatrogenic ocular surface complications require a multidisciplinary approach involving early diagnosis, personalized treatment strategies, and targeted postoperative care. Comprehensive pre- and postoperative planning is essential to optimize both visual function and long-term ocular surface integrity, ultimately ensuring a balance between oncologic control with functional and aesthetic preservation.

## Simple Summary

This review comprehensively examines iatrogenic ocular surface complications following surgical and adjunctive treatments for ocular tumors. It highlights the pathophysiological mechanisms, diagnostic challenges, and therapeutic approaches for managing conditions such as eyelid malposition, symblepharon, conjunctival scarring, and exposure keratopathy. A multidisciplinary approach involving reconstructive surgery, ocular surface rehabilitation, and adjunctive therapies is essential to optimize patient outcomes and prevent long-term sequelae.

## 1. Introduction

Ocular tumors exhibit diverse and potentially severe characteristics and are primarily classified into two major categories: congenital and acquired lesions. These tumors are considered rare and vary from benign lesions to aggressive malignancies, each presenting significant risks to vision and life and requiring tailored treatment strategies. The management of ocular tumors often necessitates a multifaceted approach, prioritizing patient survival, followed by vision maintenance and functional ocular preservation. This incorporates surgery, radiation therapy, and chemotherapy. While these treatments are essential for tumor control, they significantly impact the ocular surface, potentially leading to severe iatrogenic complications [1].

The ocular surface itself is a finely regulated complex system consisting of cornea, conjunctiva, eyelids, and tear film. It serves as a critical interface between the eye and the environment, providing anatomical, physiological, and immunological protection [2]. Acute and chronic complications consequent to ophthalmic tumor management are multifactorial. Both surgery and adjunctive radiotherapy and chemotherapy can impair the ocular surface, affecting the lacrimal system, eyelid functionality, and corneal–conjunctival integrity [3]. As a matter of fact, ocular surface disturbances resulting from surgical procedures can lead to various complications, including dry eye disease (DED), corneal impairment ranging from epitheliopathy to corneal ulcers, limbal stem cell deficiency (LSCD), and conjunctival involvement from scarring to severe symblepharon. It is important to emphasize the need for early ocular surface disease diagnosis, which can prevent the onset of a vicious cycle of worsening symptoms that become harder to manage over time. A comprehensive ophthalmic examination is fundamental, including slit lamp examination, with the eversion of the upper and lower eyelids, tear film break-up time (BUT), tear meniscus height, meibomian gland secretion quality and loss, bulbar redness, and blink rate [4]. Additionally, the ocular surface can be evaluated through non-invasive diagnostic devices. Also, lipid layer analysis serves as a valuable tool for tailoring treatment strategies [5]. The therapeutic management of ocular surface disease extends beyond symptomatic relief focusing on para-inflammatory responses. Core strategies include the use of artificial tear substitutes to enhance lubrication, often supplemented by targeted anti-inflammatory therapies such as corticosteroids, cyclosporine A, or lifitegrast, which modulate immune-mediated inflammatory pathways by reducing cytokine-mediated epithelial and goblet cell damage [6]. Also, Vitamin A ointments support epithelial regeneration and stabilize the tear film, especially in cases associated with severe epithelial damage or goblet cell loss. Treatments for severe or refractory DED include autologous serum eye drops and platelet-rich plasma, both of which deliver bioactive factors that support epithelial repair and modulate inflammation. Topical insulin has also demonstrated the potential to promote epithelial regeneration [7]. In more severe cases, amniotic membrane transplantation provides an anti-inflammatory and anti-fibrotic substrate that promotes epithelial healing and minimizes scarring [8].

This review explores the complexities of therapeutic approaches for ocular and adnexal tumors, analyzing the impact of these interventions on the ocular surface and emphasizing the need to balance effective tumor management with ocular surface preservation.

## 2. Materials and Methods

This narrative review was conducted by performing a comprehensive literature search on PubMed to identify relevant articles on the topic of interest. A structured search strategy was developed to ensure a search strategy with different keywords was developed to ensure broad coverage of the literature (Table 1).

**Figure 1:**
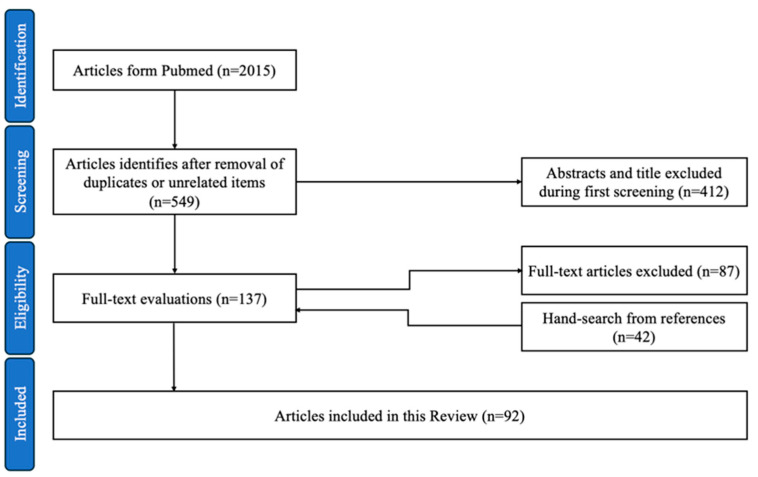
Flowchart for the article selection process.

The search was conducted in January 2025 and included reviews and clinical studies in the English language, with no restrictions on publication date. A total of 2015 full-text articles were identified on PubMed. After removing duplicates and unrelated items, 549 articles were selected for abstract screening. Following this step, 137 full-text articles were evaluated for inclusion, with 87 studies excluded due to irrelevance. A manual search of the reference lists from the included studies identified 42 additional relevant sources, bringing the total number of articles included in this review to 92 (Figure 1).

## 3. Discussion

### 3.1. Tumors of the Eyelids

The primary challenge in oculoplastic surgery extends beyond tissue removal. In order to enhance patient and surgeon satisfaction, eyelid reconstruction aims to ensure an adequate radicality of the procedure while preserving eyeball protection, visual field, and facial aesthetics. Adjuvant radiotherapy or topical chemotherapy is often employed to minimize the risk of tumor recurrence [1]; however, it heightens the risk of post-surgical complications, underscoring the need for careful preoperative and postoperative planning to optimize outcomes. A detailed preoperative evaluation of eyelid static and dynamic functions is essential to guide the use of autologous flaps and grafts, ensuring optimal reconstructive outcomes and allowing intraoperative adjustments to address unforeseen challenges. Given the functional interplay between the eyelids and the ocular surface, the direct manipulation of periocular tissues during surgical management should be approached with caution, as eyelid function ensures the maintenance of the tear film’s composition and distribution [9]. Surgical interventions, particularly in cases of extensive resection, carry the risk of complications such as eyelid malposition or dynamic abnormalities, which require individualized evaluation and targeted management [10]. To mitigate these risks, reconstructive techniques, primarily designed to achieve proper oncologic radicality, are also designed to preserve eyelid function. In this context, flaps and grafts remain the cornerstone strategies for addressing large full-thickness defects. Based on the clinical characteristics of the tumor, patient history, and its location on the eyelid, different surgical approaches may be considered.

#### 3.1.1. Tumors of the Eyelids: Clinical Entities

##### Basal Cell Carcinoma

**Figure 2:**
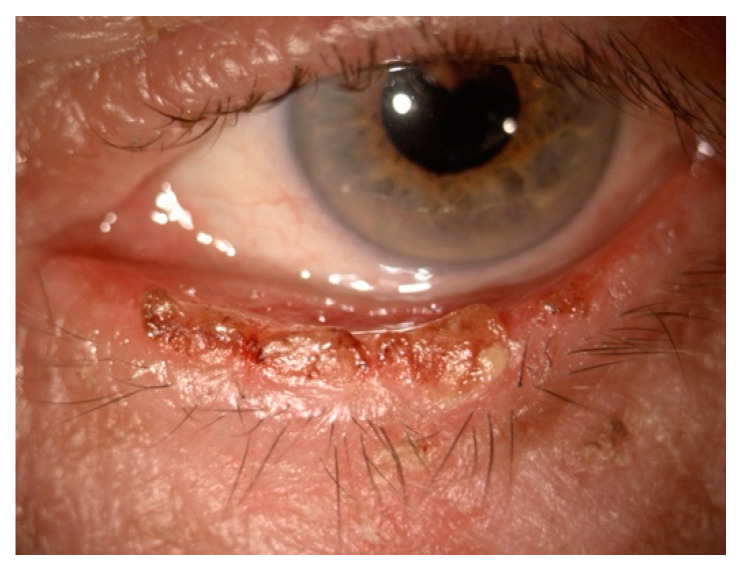
A 79-year-old patient with an ulcerative lower lid lesion with the structural distortion of the eyelid margin in the left eye, histopathologically proving to be a basal cell carcinoma.

Basal cell carcinoma (BCC) is the most common malignant eyelid cancer, accounting for 75–90% of skin cancers and 90% of eyelid malignancies in the United States [11]. It is hypothesized to originate from the basal cells of the epidermis and most frequently occurs on the lower eyelid, followed by the medial canthal area and the upper eyelid (Figure 2).

For well-localized BCC, despite the fact that several alternatives such as cryosurgery, curettage, electrocautery, radiotherapy, and chemotherapy have been used, the preferred treatment method in clinical practice has been considered surgical removal using Mohs micrographic surgery or broad local excision with intraoperative control of margins, followed by eyelid reconstruction [11,12]. Locally advanced periocular BCC, which occurs in 5–17% of the cases, is deemed unresectable due to its infiltration into deeper anatomical structures, including the orbit, nasolacrimal system, paranasal sinuses, nasal cavity, periosteum, bones, and brain [12].

##### Squamous Cell Carcinoma

**Figure 3:**
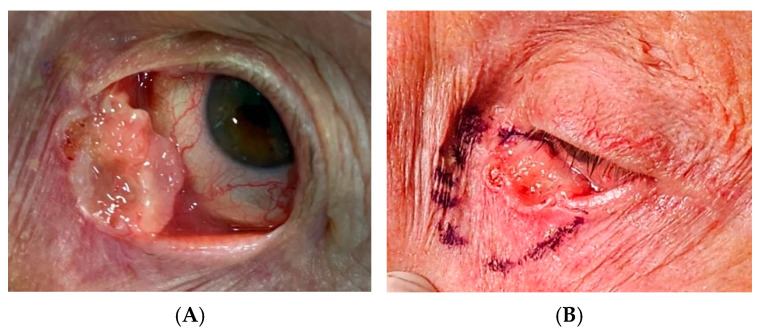
Preoperative (**A**) and intraoperative (**B**) images of a squamous cell carcinoma of the lower eyelid. The tumor appears as an irregular, ulcerated, and infiltrative lesion affecting the inner eyelid margin and extending into the tarsoconjunctival plane. Surgical excision with wide margins was performed.

Squamous cell carcinoma (SCC) affecting the eyelid and surrounding skin ranks as the second most frequent type of periocular cancer, accounting for 5–10% of the cases, following basal cell carcinoma [13]. SCC is characterized by aggressive local invasion and potential for perineural spread, increasing the risk of recurrence and requiring careful surgical planning (Figure 3).

**Figure 4:**
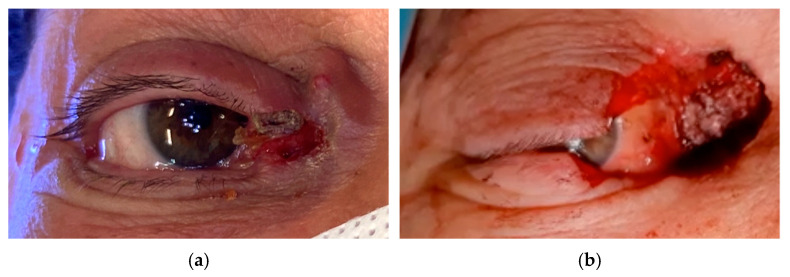
(**a**) Preoperative image of a 56-year-old patient with a squamous cell carcinoma of the medial canthus of the right eye, presenting as a firm, ulcerated lesion with infiltrative margins and rapid growth over a two-week period; (**b**) intraoperative image showing periosteal involvement. Partial tumor excision was performed, followed by reconstruction using a glabellar flap in combination with a cheek flap for adequate tissue coverage and functional restoration. (Courtesy of Di Maria Alessandra, MD).

The established treatment protocol for periocular SCC involves surgical excision with controlled margins, often utilizing the ‘no-touch technique’ which minimizes the risk of tumor seeding and enhances oncological safety. Surgical resection for extensive lesions may necessitate careful postoperative monitoring and possible secondary reconstructive intervention [14]. Given regional lymphatic spread potential, staging of at least the first-tier lymph nodes, particularly in the cervical region, is recommended to assess for metastatic disease [15]. In the case of advanced or recurrent SCC with orbital invasion, orbital exenteration may be required, and reconstruction strategies are implemented to restore volumes (Figure 4) [15].

Additionally, postoperative scar retraction, chronic pain, and minor volume deficits, particularly in irradiated or extensively resected regions, are frequent concerns. Autologous fat grafting has emerged as an effective method for addressing these issues by restoring lost volume and mitigating radiation-induced fibrosis. This technique has shown promising results in patients who underwent enucleation or exenteration for periocular malignancies, enhancing both functional and aesthetic outcomes [16].

##### Ocular Adnexal Lymphoma

Ocular adnexal lymphoma (OAL), primarily represented by low-grade mucosa-associated lymphoid tissue (MALT) lymphoma (50–90%), requires molecular diagnostics to refine the differential diagnosis and guide targeted therapeutic strategies [17]. Hodgkin’s lymphoma rarely impacts ocular adnexal structures unless there is extensive systemic spread or a prior history of systemic Hodgkin’s lymphoma. For stage 1 MALT lymphoma, Tanimoto and colleagues reported excellent local management with biopsy or surgical excision alone. Although these methods are not curative, they effectively control the local progression of the disease, with 69% of the patients requiring no further treatment over a median follow-up of 7.1 years and overall survival rates of 94% and 71% at ten and fifteen years, respectively [18]. Systemic therapy remains necessary for disseminated disease, and close follow-up is essential to detect recurrence, histologic transformation, or systemic progression [17,19]. Prognosis varies by site, with higher rates of systemic progression for orbital (35%) and eyelid lymphomas (70%) compared to conjunctival involvement [19]. For localized cases, external beam radiation therapy (EBRT) is considered the standard of care [17]. However, postoperative complications following radiotherapy may include dry eye disease, meibomian gland dysfunction, and, in more extensive cases, keratitis or exposure-related epithelial damage. These effects are dose-dependent and may be mitigated by lid-sparing techniques and proactive ocular surface management.

##### Merkel Cell Tumor

**Figure 5:**
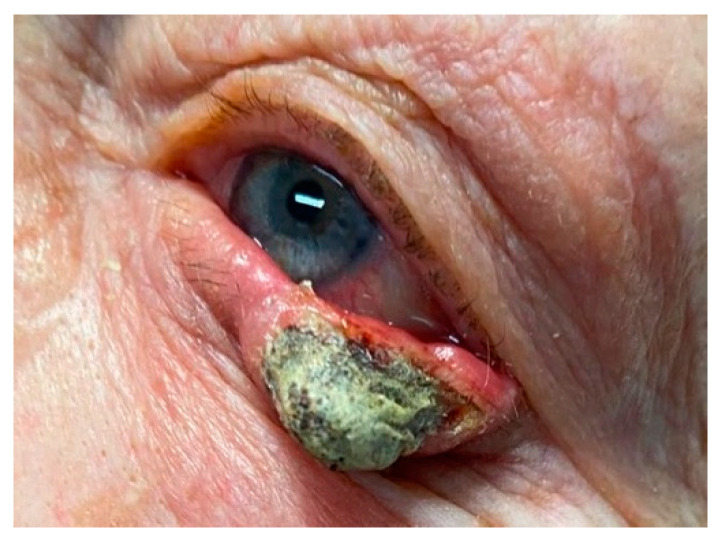
Advanced Merkel cell carcinoma of the eyelid in an 83-year-old woman, presenting as a firm, nodular lesion with ulceration, necrotic crusting, and local tissue invasion. Histopathology confirmed Merkel cell carcinoma with characteristic neuroendocrine features. (Courtesy of Di Maria Alessandra, MD).

Merkel cell carcinoma (MCC) is a rare and aggressive neuroendocrine malignancy of the skin, with a notable predilection for the periorbital region (Figure 5).

It accounts for a small fraction of cutaneous malignancies but is characterized by a high propensity for local recurrence, regional lymph node involvement, and distant metastasis. Clinically, MCC often presents as a rapidly enlarging, painless, firm nodule, which may exhibit a reddish or violaceous hue. Due to its nonspecific appearance, it may be misdiagnosed as benign lesions such as chalazion or cysts, potentially leading to delays in appropriate management [20,21].

#### 3.1.2. Surgical Management of Eyelid Tumors: Overview of Flap and Graft Techniques

Flap techniques (e.g., sliding, advancement, rotation, and transposition) are tailored to the post-surgical defect’s specific location, size, and depth. These local skin or musculocutaneous flaps are preferred in periorbital soft tissue repairs for their superior ability to match the texture and color of the surrounding skin [22]. Reconstructive flap techniques, such as the Hughes and Cutler–Beard procedures, are indicated for upper and lower eyelid defects involving more than 50% of the lid margin, offering functional restoration and improved aesthetics [22,23]. Additional techniques, including Mustardé cheek rotation flap and Tenzel semicircular rotational flap, target lateral eyelid defects, utilizing adjacent skin and muscle to preserve eyelid contour and function with minimal tension. The Fricke flap by utilizing temporal skin is often chosen for optimal color and texture compatibility, vital for both functional and cosmetic restoration. These flaps are designed not only to close the defect but also to ensure continued ocular protection and tear film distribution postoperatively.

Grafts, in contrast, are indispensable for reconstructing defects where local tissue is insufficient or unsuitable. Commonly used graft types include full-thickness skin grafts, composite grafts, and mucosal grafts, each chosen based on the specific reconstructive needs. Composite grafts, combining skin and cartilage, are particularly useful for reconstructing defects that require structural support, such as in the lower eyelid. Mucosal grafts from the oral cavity or nasal septum may be utilized for conjunctival surface restoration, addressing cases where scarring or inflammation compromises ocular surface integrity [24,25].

#### 3.1.3. Iatrogenic Ocular Surface Complication Following Eyelid Tumor Surgery

##### Postoperative Inflammatory Response of the Ocular Surface

Localized inflammatory responses triggered by eyelid neoplasm management can exacerbate immune-mediated inflammatory response, disrupting tear production, ocular surface integrity, and epithelial homeostasis [26,27]. Additionally, radiation therapies for periocular malignancies may contribute to inflammation and cellular stress, further compounding the risk of epithelial barrier dysfunction [1]. Even when no visible postoperative structural changes occur, the secretory function of the tear film must be assessed, as iatrogenic conditions like blepharitis and glandular dysfunctions must be identified and treated to protect the ocular surface [28]. On top of this, iatrogenic inflammation driven by fibroblast activation and collagen deposition plays a key role. At the cellular level, immune cells including macrophages and monocytes secrete cytokines and growth factors essential for epithelial repair and maintenance [6]. In addition, radiotherapy- and chemotherapy-related inflammation further compromise tissue regeneration [29]. Radiation therapy can lead to ocular surface disorders such as DED, keratitis, and conjunctivitis due to its high-energy impact on cellular structures, leading to cell death and reduced gland function [29]. Moreover, hormonal imbalances induced by systemic chemotherapy can also disrupt the eyelid’s glandular function, manifesting as DED, blepharitis, and meibomian gland dysfunction. This is especially evident with the use of selective estrogen receptor modulators and aromatase inhibitors, where such side effects are linked to these hormonal disruptions [30]. Moreover, anatomical factors may also predispose patients to ocular surface complications; for instance, individuals with proptosis are at higher risk of developing DED postoperatively due to increased ocular surface exposure and incomplete eyelid closure [31].

##### Lagophthalmos

Iatrogenic lagophthalmos resulting from the surgical excision of periorbital tumors can manifest as either transient or permanent depending on the extent of tissue removal, nerve involvement, and postoperative complications [32]. Transient lagophthalmos often arises from temporary factors such as postoperative flogosis and edema, mild VII nerve impairment, iatrogenic orbital septal displacement, or local anesthesia. It resolves as the healing process progresses [32]. Permanent lagophthalmos typically result from excessive removal of eyelid tissue, irreversible nerve damage, radiation-associated fibrosis, or inadequate postoperative care. Such conditions can lead to infections, significant scarring, and fibrosis, which may further result in visual loss [33].

Lagophthalmos should be evaluated using corneal fluorescein staining during a slit-lamp examination to accurately assess its impact on the ocular surface [32,34,35]. Ocular surface complications arising from lagophthalmos range from mild symptoms of DED to more severe conditions such as chronic exposure keratopathy and corneal abrasions. Over time, these issues may lead to persistent epithelial defects, ulceration, microbial keratitis, and corneal perforation [36].

Management of Transient and Permanent Lagophthalmos

The management of lagophthalmos, both transient and permanent, employs a spectrum of medical and surgical interventions tailored to the underlying causes of exposure keratopathy and aimed at preserving the ocular surface. For transient lagophthalmos supportive treatments such as the application of preservative-free artificial tears, ointments, hydrating drops, and nighttime eyelid taping are vital [36,37]. These measures help maintain the tear film and protect against corneal exposure, particularly in patients with suboptimal Bell’s phenomenon, which can lead to corneal desiccation and potential visual impairment [33]. Temporary tarsorrhaphy may also be used for short-term management, partially closing the eyelids to protect the cornea while allowing for assessment and application of treatments [38]. In more severe or permanent cases, surgical revision may be necessary. This includes procedures to address anterior lamellar deficiencies through scar release and interpositional skin grafting, or more extensive measures such as the implantation of gold or platinum weights in the upper eyelid to enhance closure via gravity, with platinum being preferred for its density and lower visibility [39]. Further surgical interventions might include the tightening of the lower eyelid via lateral tarsal strip (LTS) procedures or the recession of retractor muscles, often supplemented with autologous grafts from the ear or nasal cartilage to elevate the eyelid adequately [33,40]. In cases of severe facial nerve paralysis, advanced reconstructive techniques such as midface elevation using autogenous fascia slings or temporalis muscle transposition are considered to reanimate the face and restore functional symmetry [37].

##### Upper Eyelid Malposition

Upper eyelid malposition is a rare but possible iatrogenic complication following eyelid tumor surgery. Depending on the underlying cause, treatment options include levator muscle elongation, levator aponeurosis reinsertion onto the tarsal plate, Müller’s muscle myectomy, or the recession of both the Müller muscle and the levator [41]. In cases of orbicularis damage, the insertion of a gold weight implant may be required to ensure proper eyelid closure and corneal protection [42]. Recently, autologous fat grafting showed promise in improving eyelid function by targeting fibrotic tissue and alleviating post-surgical pain, thereby enhancing aesthetic outcomes [16].

##### Lower Eyelid Malposition

Lower eyelid malposition is a common postoperative complication following tumor excision, encompassing conditions such as entropion, ectropion, and scleral show, with potential impact on both function and aesthetics [43]. Preventive strategies include meticulous dissection, effective hemostatic approaches, and strategic surgical planning [44]. Additionally, the minimization of orbicularis muscle manipulation and canthal tendon support during skin–muscle flap suspension can further minimize postoperative conjunctival chemosis and consequent ocular surface disruption [45]. Nevertheless, cicatricial and gravitational forces may still compromise eyelid structural integrity due to adhesions of the orbital septum, excessive removal of skin or muscle, lid laxity, or inadequate lid suspension [46].

Entropion, Ectropion and Round Eye

Entropion is characterized by the inward turning of the eyelid margin, which brings eyelashes and skin into contact with the cornea, potentially causing irritation, abrasion, and corneal ulceration. This condition often stems from surgical trauma involving the excessive removal or manipulation of eyelid tissues, which leads to scar formation and contracture, disrupting the eyelid’s normal tension [47]. The complication may be further exacerbated by inadequate surgical reconstruction, orbital septum mishandling, incorrect reinsertion of the orbicularis oculi muscle, or facial nerve (cranial nerve VII) injury [48].

It is crucial to differentiate scleral show, clinically defined as round eye, from ectropion. Ectropion is characterized by the outward turning of the eyelid margin, resulting in exposure of the palpebral conjunctiva, often accompanied by pronounced scleral show (more than 3 mm). This malposition results in DED, ocular surface irritation, and conjunctival epithelization [36]. Moreover, epiphora is frequently observed as a result of lacrimal punctum displacement and reflex tearing triggered by corneal exposure [49,50].

**Figure 6:**
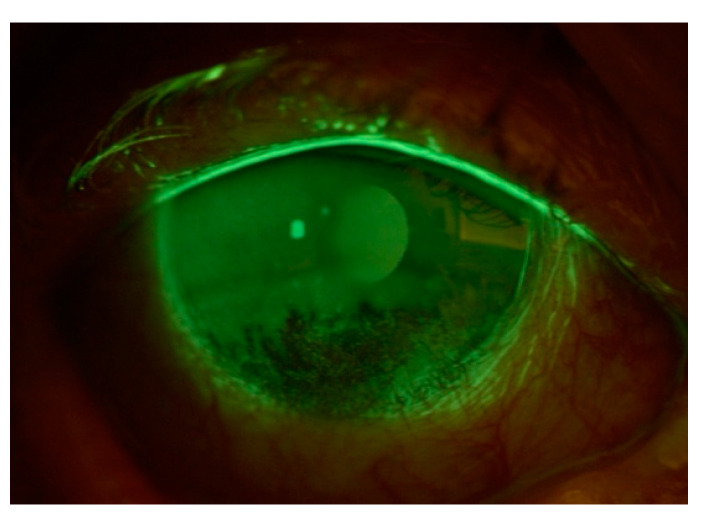
Exposure keratopathy secondary to lower eyelid malposition in the right eye of an 86-year-old male. The fluorescein staining highlights areas of punctate epithelial cell loss, indicating compromised ocular surface integrity and inadequate tear film coverage.

Round eye is an anatomical condition where the sclera is visibly exposed beneath the eyelid margin for less than 3 mm. In this condition, the eyelid margin remains adherent to the globe, and unlike ectropion, there is no eversion of the eyelid margin, and epiphora is typically absent [51]. Still, the associated corneal exposure may still contribute to DED (Figure 6) [10].

The development of ectropion and round eye is primarily due to factors that induce inferior vertical traction and the rounding of the lateral canthus. These include damage to the orbicularis oculi muscle; excessive removal of skin, fat, or muscle from the lower eyelid; and both anterior and posterior lamella shortening. Scar entrapment and damage to the eyelid’s supportive tendons, coupled with muscle hypotony, also contribute to the outward turning of the eyelid margin [10,50]. Furthermore, the laxity of lower eyelid structures, often influenced by gravitational forces, exacerbates these conditions. Preventive strategies rely on effective surgical planning and the strategic use of flaps to avoid excessive skin removal and the over-shortening of the anterior lamella [49,50].

Management of Entropion and Ectropion

- Management of Entropion

For mild to moderate cases of lower eyelid iatrogenic entropion, tarsal fracture surgery is a commonly used method. This procedure involves creating a controlled break in the tarsal plate to correct the inward turning of the eyelid margin [52]. Upper eyelid cases may require anterior lamellar repositioning or tarsal wedge resection to elongate the posterior lamella and restore anatomical alignment [53]. In more severe forms, posterior lamellar grafts, either autologous or synthetic, are used to replace or augment deficient tissues, thereby providing structural support to the posterior lamella [32,34]. When the cicatricial entropion of the upper eyelid involves significant scarring or tarsal thinning, anterior lamellar repositioning is often the first step; however, if posterior eyelid margin keratinization or profound tarsal plate structural loss is present, more complex reconstruction such as posterior approach or tarsal wedge resection may be required [35].

- Management of Ectropion and Round Eye

**Figure 7:**
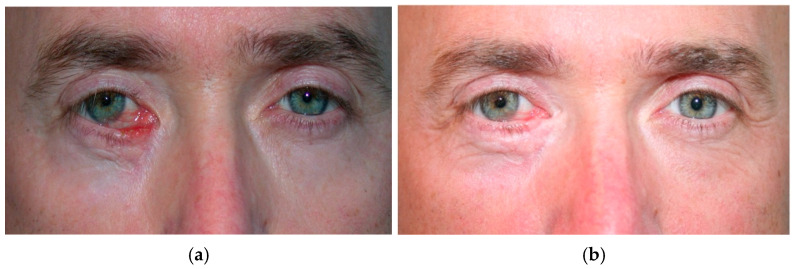
(**a**) Cicatricial ectropion following squamous cell carcinoma excision of the lower eyelid in a 55-year-old patient, previously treated at another center with anterior lamellar lengthening via a pedicled rotational flap from the upper eyelid, resulting in persistent medial cicatricial retraction, epiphora, and tissue rigidity. (**b**) To address these cicatricial sequelae, Coleman lipostructure was performed. Adipose tissue was harvested from the infraumbilical region under local anesthesia, processed by centrifugation (3000 rpm for 5 min), and injected into the dermo-hypodermic junction of the scarred area (0.5 mL) using an 18-gauge cannula with the snap-on wing technique. One-year follow-up showed a subjective reduction in epiphora, residual lagophthalmos resolution, and significant improvement in skin texture. (Courtesy of Di Maria Alessandra, MD).

Management strategies for iatrogenic ectropion differ based on the timing of intervention. In the immediate postoperative phase, minor ectropion can be managed conservatively with techniques such as suspension frost suture placement, temporary tarsorrhaphy, revisional canthopexy within the first two weeks, and lateral massage or skin taping to support the eyelid in a vertical–lateral direction [54,55]. Intralesional steroid injections may also be used to soften scarring and promote tissue remodeling. If ectropion persists beyond six to eight months, despite conservative measures, surgical intervention is typically recommended [56]. Techniques such as LTS or canthoplasty (often combined with support grafts), medial spindle, or scar revision are commonly employed to address horizontal laxity and restore both functionality and aesthetics [43,57]. Moreover, autologous fat grafting, or lipostructure, offers a promising solution for correcting cicatricial ectropion, especially in cases refractory to traditional procedures (Figure 7) [16,58,59]. In both round eye and ectropion, excessive scleral show increases evaporative tear loss, and tear substitutes with properties designed to delay evaporation and enhance hydration are recommended.

When upper cicatricial ectropion results from tarsal tension due to excessive skin removal, correction typically involves skin grafting or orbicularis oculi flap rotation from the same lid [23,60,61]. Additionally, posterior lamella elongation using cartilage or hard palate mucosa may be integrated with external canthopexy to further stabilize and correct the eyelid structure [49,61]. Further enhancing outcomes, the Reidy–Adamson flap technique involves anchoring a triangular flap of the lateral orbicularis muscle to the periosteum of the upper orbital rim, proving effective in managing the vertical and horizontal laxities often unaddressed by traditional lateral canthal tightening. Moreover, the strategic use of lower lateral dermal flaps, as described by Milbratz-Moré, further refines both functional and aesthetic outcomes [50,56,62].

##### Epiphora

**Figure 8:**
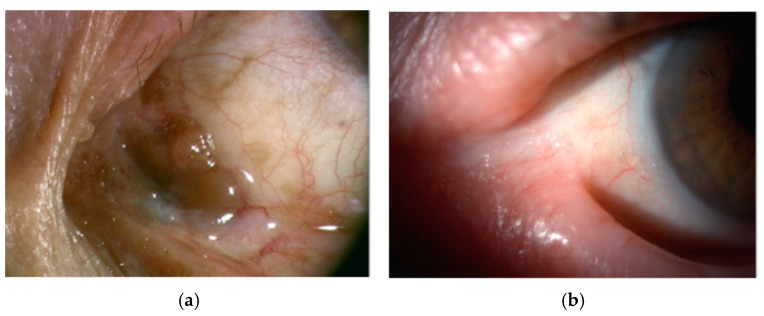
(**a**) Caruncular melanoma with fornical conjunctival involvement. Surgical excision typically extends to adjacent cutaneous structures, including the eyelid margin, to achieve oncologic control. (**b**) Postoperative sequelae in a different patient illustrating one of the potential complications following caruncular surgery. Conjunctival adhesions have led to ankyloblepharon, lacrimal punctum malposition, and epiphora.

DED most commonly arises from reduced production of the lipid layer, leading to destabilization and increased evaporation of the tear film [63]. Ectropion also contributes to lacrimal functional issues such as epiphora and DED due to the malposition of the lacrimal punctum relative to the lower eyelid fornix. This misalignment, which may result from conjunctival adhesions or cutaneous traction-induced cicatricial ectropion, disrupts the lacrimal pump mechanism, leading to insufficient tear drainage and subsequent dryness despite the presence of excessive tearing (Figure 8) [64].

The management of these conditions involves strategic interventions such as eyelid repositioning and lacrimal pathway reconstruction, which aim to correct mechanical dysfunctions that impair tear distribution or exacerbate tear film instability. Moreover, surgical interventions should prioritize the precise repositioning of the lower lacrimal punctum against the globe to maintain the lacrimal pump mechanism, thereby preventing tear drainage dysfunction and associated symptoms such as epiphora or DED. This process typically involves the insertion of a Bowman probe to preserve punctal patency, followed by the excision of a small diamond-shaped segment of inferior conjunctival tissue near the lower lacrimal punctum to facilitate its realignment. These steps are carefully executed before performing lateral tarsal strip (LTS) surgery to ensure optimal anatomical restoration and functional outcomes [65,66]. Moreover, in patients undergoing long-term treatment with topical prostaglandins, the strategic insertion of a lacrimal punctum stent mitigates fibrosis-related complications, thereby sustaining gravitational tear outflow [67].

##### Exposure Keratopathy, Corneal Ulcers, and Infections

**Figure 9:**
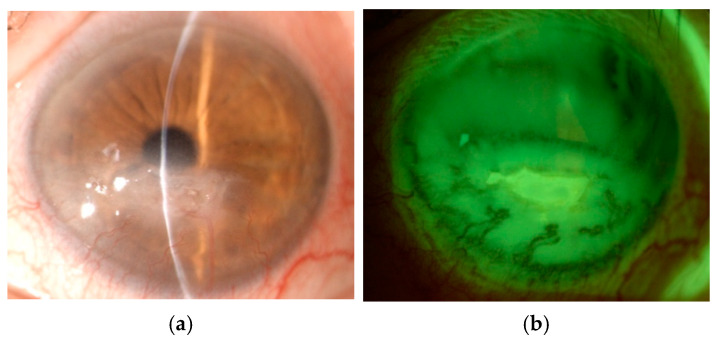
(**a**) Severe exposure keratopathy complicated by corneal ulceration of the left eye in a 78-year-old patient following iatrogenic lagophthalmos. The cornea exhibits significant epithelial breakdown, stromal thinning, and corneal neovascularization. (**b**) Fluorescein staining demonstrating an extensive epithelial defect with positive fluorescence.

Eyelid malposition and lagophthalmos expose the ocular surface to environmental stressors, contributing to tear film instability, increased evaporation of the tear film, and mechanical trauma. These lead to the development of exposure keratopathy, characterized by epithelial defects, corneal thinning, and, in severe cases, ulceration or perforation (Figure 9) [68].

Also, altered eyelid anatomy creates a predisposition for microbial colonization and subsequently to infections. The compromised tear film and epithelial barrier, coupled with prolonged exposure, facilitate the entry of pathogens, leading to conditions such as bacterial keratitis or conjunctivitis [69]. This risk may be further exacerbated by poor blink dynamics, which impair the distribution of the tear film and the clearance of microorganisms [70]. The goal of treatment is to increase tear volume on the ocular surface [7,64,69,71]. This can be achieved through the administration of exogenous tear substitutes or by enhancing autologous tear production by reducing lacrimal loss. Punctal plugs can be beneficial as they block tear drainage into the lacrimal system, thereby preserving moisture on the ocular surface [70]. Additionally, topical anti-inflammatory agents such as cyclosporine A or lifitegrast help to reduce ocular surface inflammation. In cases of corneal epithelial impairment, the introduction of topical antibiotic therapy is necessary to minimize the risk of corneal infections [4,64,71]. Other mechanical approaches include nocturnal eyelid taping to reduce corneal exposure during sleep, thereby helping to slow the progression of keratopathy [70,71].

##### Dry Eye Disease After Eyelid Tumor Management

Dry-eye syndrome often exacerbates after eyelid surgeries due to mechanical alterations at the corneoscleral and conjunctival interface. Mechanical changes following surgery potentially unveil subclinical conditions of ocular surface instability, contributing to increased tear evaporation or reduced tear production [72]. This condition manifests with symptoms such as ocular discomfort, red eye, foreign body sensation, blurred vision, photophobia, and ocular pain, and increases the risk of corneal abrasion and ulceration [63]. Patients with proptosis, exophthalmos, or horizontal lid laxity are inherently more susceptible to developing DED postoperatively, particularly those with a history of refractive surgeries like LASIK, where terminal corneal nerves are often transected, impairing reflex tearing [73,74,75]. Also, eyelid laxity impairs the necessary muscular pressure on the meibomian glands, essential for their proper function, leading to compromised meibum secretion. This deficiency is exacerbated by inefficient blinking, which allows for the accumulation of toxins, pathogens, and foreign bodies on the ocular surface. Furthermore, reduced blink rates and incomplete blinks are linked to significant alterations in tear film stability and overall ocular surface health [76]. Such irregular blinking patterns disrupt the lipid layer of the tear film, which is crucial for reducing tear evaporation and maintaining the integrity of the tear film. This disruption can result in increased tear film instability and contribute to various ocular surface disorders [70,77]. As already mentioned, the etiology of DED often stems from an alteration in the lid margin, resulting in a misalignment with the globe. This misalignment disrupts the normal function and structure of the tear menisci, adversely affecting the distribution of the tear film and the spread of the lipid layer. Such disruptions can alter the qualitative composition of the tears, thereby heightening the risk of ocular inflammation and infections [28].

A 2012 study investigated the impact of full-thickness eyelid reconstruction on dry eye disease using advanced thermographic assessment (Tomey TG-1000, Tomey Corp., Nagoya, Japan) in patients who underwent Hughes or Cutler–Beard bridge flaps. The cohort included 17 individuals, with postoperative follow-up ranging from 3 to 63 months. Ocular surface temperature, used as a proxy for tear film stability, showed no significant differences between the reconstructed eyes and healthy controls. Likewise, Schirmer’s test and tear break-up time revealed comparable outcomes, suggesting that such reconstructive procedures do not significantly impair tear film function in the long term [78].

Dry Eye Syndrome Management

Postoperative management of DED begins with ocular surface lubrication, which remains a first-line intervention to stabilize the tear film and alleviate symptoms. Recovery is often gradual, requiring weeks to months, underscoring the need for early intervention. For persistent cases, punctal plugs help maintain adequate tear volume, while anatomical contributors may necessitate surgical correction through procedures like the repositioning of the lower eyelid, canthoplasty, or midface suspension [79]. Also, cyclosporine 0.05% emulsion was the first medication approved by the US FDA for managing this condition. Since then, additional agents such as lifitegrast and cyclosporine 0.09% have also gained approval. These immunomodulatory therapies enhance tear production by reducing conjunctival inflammation cells and promoting goblet cell-mediated mucin secretion. Clinical trials have consistently shown cyclosporine to be more effective than placebos, markedly reducing corneal staining and enhancing Schirmer scores [80]. When combined with punctal plugs, these treatments effectively maintain moisture on the ocular surface and foster long-term ocular health [81].

A comprehensive overview of the most frequent iatrogenic ocular surface complications following eyelid tumor surgery is summarized in Table 2.

### 3.2. Tumors of the Conjunctiva

Conjunctival tumors encompass a variety of melanocytic and nonmelanocytic lesions, either benign or malignant. The most common conjunctival cancers are represented by melanocytic lesions and OSSN [82,83,84,85].

#### 3.2.1. Tumors of the Conjunctiva: Clinical Entities

##### Melanocytic Tumors

**Figure 10:**
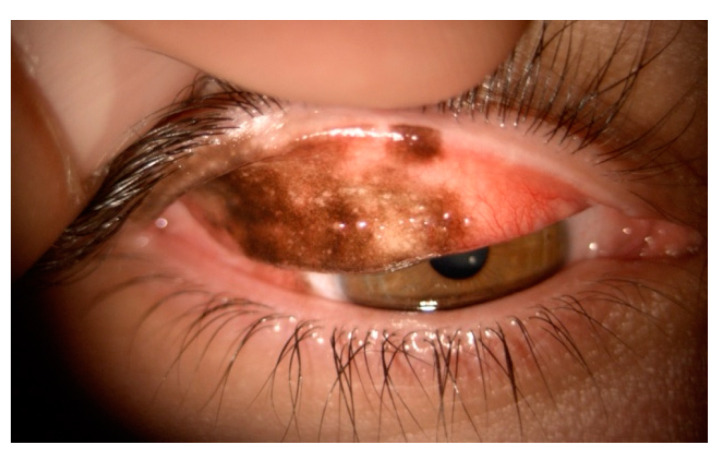
Primary acquired conjunctival melanosis (PAM) in the right eye of a 34-year-old male patient. The lesion appears as an irregularly pigmented area with indistinct margins, involving the bulbar conjunctiva. Clinical monitoring is essential to assess potential progression to conjunctival melanoma.

Melanocytic tumors of the conjunctiva comprise a range of entities including conjunctival nevi, complexion-associated melanosis, primary acquired melanosis (PAM), and invasive conjunctival melanoma (CoM) (Figure 10) [83,86,87,88,89].

Diagnostic challenges for conjunctival melanocytic lesions are many. This is linked to the small size of typical conjunctival biopsies, potential tangential sectioning during embedding, and the frequent presence of crush artifacts, complicating histopathological evaluation [90]. Additionally, the morpho-phenotypic plasticity of melanocytic tumors, as observed in dedifferentiated melanoma variants, adds further challenge to histopathological assessment, necessitating comprehensive immunohistochemical complexity to their diagnostic assessment [91].

Conjunctival Melanoma

CoM is a notably rare malignant tumor, with an incidence of 0.2–0.7 cases per million in Europe and the USA, representing only 1.6% of the non-cutaneous melanomas and less than 5% of the ocular melanomas, with most arising from the uveal tract. For localized disease, the standard treatment includes surgical excision using the ‘no-touch technique’ with at least 3–4 mm clear margins, complemented by adjuvant double freeze–thaw cryotherapy to minimize the risk of recurrence [86,92,93,94]. The choice of reconstructive technique depends on the extent of the surgical defect: small lesions may be managed with primary conjunctival closure, whereas larger or more complex ones may require buccal, mucous membrane, or amniotic grafts. If the surgical defect post-melanoma removal is small, primary closure of the conjunctiva is feasible. However, larger defects might necessitate conjunctival reconstruction using grafts such as buccal mucosa, mucous membrane, or amniotic membrane [92].

Adjunctive treatments include topical chemotherapy with mitomycin C, particularly when surgical margins are positive for intraepithelial disease, and topical interferon alpha 2b, which has been used for managing primary acquired melanosis with atypia and conjunctival melanoma, with or without concurrent cryotherapy [93]. When histology confirms invasive disease or margin positivity, re-excision [90] or adjuvant proton therapy [95,96] may be considered, as topical agents have limited penetration [96,97]. In cases of advanced or unresectable tumors, enucleation or exenteration followed by radiation may be necessary [90]. Postoperatively, repeated autologous fat grafting has demonstrated efficacy in managing painful scar syndromes in the periocular region, and in alleviating discomfort in irradiated orbits following enucleation and orbital exenteration [15,16]. For locally advanced or metastatic disease, systemic therapies including chemotherapy, immunotherapy, and targeted inhibitors of the MAPK and BRAF pathways are employed [98]. Immune checkpoint inhibitors targeting PD-1/PD-L1 have also demonstrated efficacy [99]. Given the high recurrence rate (33–61%), coupled with a significant risk of postoperative complications, such as vision loss, scarring, limbal stem cell failure, and non-healing ulcers, life-long monitoring and individualized therapeutic strategies, guided by immunoprofiling, are essential for optimal outcomes [100].

##### Ocular Surface Squamous Neoplasia

OSSN encompasses pathological entities characterized by the dysplastic alteration of the squamous epithelium of the conjunctiva and the cornea with variable degrees of invasiveness, from mild epithelial dysplasia to conjunctival intraepithelial neoplasia in situ (CIN), and invasive squamous cell carcinoma (SCC) [13,88,101,102]. OSSN is the most common non-melanocytic tumor of the ocular surface, typically non-pigmented, though pigmented variants may occur [103]. Ultraviolet light exposure, human immunodeficiency virus, exposure to petroleum products, heavy cigarette smoking, advanced age, and male gender have been identified as significant contributors [102]. Although human papillomavirus (HPV) has been implicated, its role remains under debate [13]. Clinically, OSSN lesions can present with various morphologies, including gelatinous, papillary, leukoplakic, or opalescent characteristics, and are often associated with neo-vascularization and the presence of feeder vessels, which supply blood to the tumor. OSSN most commonly originates at the limbus and is typically found in areas with significant UV light exposure, such as the temporal and nasal interpalpebral fissures. Though usually localized and slow-growing, it carries the potential to invade deeper ocular structures such as the orbit, sinuses, or globe [104]. Diagnosis relies on histopathology and management often begins with surgical excision.

Ocular Surface Squamous Neoplasia Management

The traditional surgical approach involves a “no-touch” technique, but recurrence rates with excision alone remain high, reaching up to 56%, even with histologically clear margins [105,106]. Extensive excision is often required for larger tumors, particularly those involving the tarsal conjunctiva or caruncle, and may be complemented by amniotic membrane transplantation to aid ocular surface reconstruction [107]. However, given the associated risk, such as symblepharon and goblet cell depletion, adjuvant therapies are routinely integrated with surgical excision to minimize recurrences and preserve ocular surface integrity [105]. In this context, over the past two decades cryotherapy and topical chemotherapy, including mitomycin C (MMC), 5-fluorouracil (5-FU), and interferon alpha-2b (IFNα2b) have proven effective in reducing recurrence rates, with IFNα2b particularly favored for its low toxicity profile [30,97,101,108]. Nonetheless, the cytotoxic effects of these drugs (immune checkpoint inhibitors, HER2 inhibitors, BCR-ABL inhibitors, and proteasome inhibitors, particularly bortezomib and so on) may alter tear film composition by antibodies or metabolic byproducts buildup, impairing its protective function and leading to further complications like epithelial breakdown [109,110].

As already mentioned, surgical resection may lead to complications such as symblepharon and conjunctival scarring, necessitating careful postoperative monitoring and possible secondary reconstructive intervention [14]. A retrospective study of 389 OSSN cases from Galor et al. identified tarsal location, positive surgical margins, and severe lesion grade as key recurrence predictors. Cryotherapy applied at surgical margins decreased recurrence from 31% to 16% over five years. Additionally, topical interferon therapy effectively lowered recurrence in patients with positive margins to levels comparable to those with negative margins. Despite advancements, surgical excision alone resulted in up to 56% recurrence, emphasizing the importance of adjunctive measures. Postoperative complications, such as limbal stem cell deficiency, were noted, highlighting the need for tailored, multimodal approaches [111]. 5-FU may cause mild ocular surface symptoms and signs, including eye redness, eyelid edema, tearing, and keratopathy [109]. MMC, on the other hand, is known to be the most aggressive topical chemotherapy, often leading to significant redness and pain, sometimes forcing patients to discontinue treatment due to severe ocular surface complications. Additionally, limbal stem cell deficiency has been reported in some cases [109,112]. Punctal stenosis has been observed following treatment with both of these eye drops; therefore, patients should be advised to perform manual punctal occlusion during eye drop instillation or consider the placement of punctal plugs. The application of petroleum jelly on the lower eyelid skin is also recommended to minimize the risk of irritation [109,110]. IFNα-2b eye drops have a very mild side effect profile and are considered the least toxic topical treatment compared to MMC and 5-FU. To alleviate potential discomfort, frequent lubrication with preservative-free artificial tears is recommended throughout the day [113].

##### Conjunctival Ocular Adnexal Lymphoma

**Figure 11:**
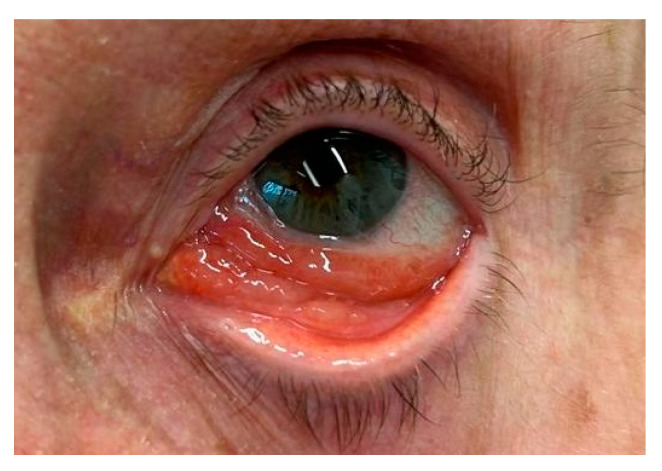
A conjunctival mucosa-associated lymphoid tissue (MALT) lymphoma, characterized by a salmon-red patch appearance and conjunctival swelling, in a 57-year-old woman. Histological examination confirmed a diagnosis of marginal zone B-cell lymphoma of MALT type. (Courtesy of Alessandra Di Maria, MD).

Specialized lymphoid tissue is present in the conjunctiva as a part of the MALT system and its role is to block antigens [19]. B-cell type non-Hodgkin lymphomas (NHLs) can also arise within the conjunctival MALT [97]. Lymphoma of the conjunctiva, a form of OAL, represents 5–10% of the extranodal lymphomas and is predominantly extranodal marginal zone B-cell lymphoma (EMZL), comprising 60% of such cases [114]. Conjunctival lymphomas often present with non-specific symptoms such as a palpable lump, irritation, mild ptosis, and a typical conjunctival fornix fleshy salmon patch (Figure 11) [19,115].

As already mentioned, conjunctival involvement generally predicts the best prognosis, with only 20% of the cases progressing to systemic lymphoma, compared to incidences of 35% and 70% for orbital and eyelid involvements, respectively [19,116]. Shields et al. reported that 85% of the patients were symptomatic, which was mild in 67% of the cases. Incisional biopsy for conjunctival lymphoma is the diagnostic gold standard, useful for both histopathological and cytological evaluation [19,117]. The initial staging of all extranodal conjunctiva lymphoma should be conducted in collaboration with a hematologist [116].

Conjunctival Ocular Adnexal Lymphoma Management

In rare cases, excisional biopsy or surgical resection may be utilized as a therapeutic approach for well-circumscribed conjunctival EMZL [19,118]. For elderly or frail patients with minimal symptoms and unilateral disease, a “wait-and-watch” approach has been suggested [118]. Radiotherapy remains the gold standard for treating lymphoma confined to the conjunctiva, classified as Ann Arbor stage 1 or T1-T2 (N0M0) according to AJCC criteria. External beam radiation therapy (EBRT) has demonstrated five-year local control rates ranging from 89% to 100% in patients with low-grade isolated OAL [117,119]. Additionally, ‘ultra-low-dose’ (or “boom-boom”) radiation therapy has been recently employed for the management of low-grade systemic lymphomas, although their application in ocular settings remains limited [120]. In selected cases of ocular adnexal lymphoma with systemic involvement, systemic chemotherapy may be warranted. Rituximab, an anti-CD20 monoclonal antibody, has been used as first-line treatment for indolent B-cell lymphomas, with improved outcomes reported when combined with chemotherapy, particularly in follicular, mantle cell, and diffuse large B-cell subtypes [19].

#### 3.2.2. Iatrogenic Ocular Surface Complication Following Conjunctival Tumor Surgery

##### Conjunctival Inflammation

Surgical procedures exacerbate inflammation and disrupt ocular surface homeostasis, sustaining the immune-driven mechanisms around DED development and progression. As a matter of fact, the conjunctival tissue of these patients shows the infiltration of CD4+ T cells and an increased expression of HLA-DR. Additionally, elevated levels of inflammatory mediators, such as intercellular adhesion molecule-1 (ICAM-1), have been identified [27]. Cyclosporine A (CsA) has been extensively studied for its efficacy in managing severe inflammatory conditions associated with DED, such as radiation-induced ocular surface disease and graft-versus-host disease [121]. Its mechanism involves suppressing T-cell activation and reducing the production of pro-inflammatory cytokines, thereby restoring tear film stability and conjunctival integrity. However, the use of cyclosporine A is frequently limited by adverse effects, particularly ocular surface discomfort. In a cohort of 35 patients with dry eye disease, 60% discontinued treatment within 12 weeks due to severe burning sensations [80].

##### Persistent Epithelial Defect

Persistent epithelial defects (PEDs) are a significant iatrogenic complication following conjunctival tumor surgery. Surgical interventions can disrupt the corneal basement membrane and epithelial integrity, promoting persistent defects [122]. They are characterized by delayed or incomplete corneal epithelial healing which persists beyond 10–14 days despite supportive therapy. PEDs result from disruptions in the corneal epithelium’s protective barrier and are exacerbated by procedures such as cryotherapy, which can impair re-epithelialization by damaging cellular and vascular integrity. PEDs increase the risk of secondary infections, stromal ulceration, scarring, and vision loss [122]. The pathogenesis usually involves an interplay of epithelial adhesion impairment, limbal stem cell deficiency, inflammation, and neurotrophic damage. Inflammation plays a critical role, with cytokines such as IL-1 and TNF-α inducing the overexpression of matrix metalloproteinases, leading to extracellular matrix degradation and stromal instability. Conditions such as lagophthalmos, trichiasis, or excessive ocular surface exposure can further exacerbate PEDs [26,122]. Management strategies must address the underlying etiology while promoting epithelial regeneration. Initial treatments include preservative-free artificial tears, bandage contact lenses, and punctal occlusion to maintain ocular hydration and protect the epithelial layer [123]. In refractory cases, treatments such as autologous serum eye drops, amniotic membrane transplantation, and scleral lenses are employed to enhance healing [124]. Novel therapies, including topical thymosin beta-4, recombinant human nerve growth factor, and growth factor-enriched formulations, have been studied to accelerate epithelial repair and provide neurotrophic support [125].

##### Limbal Stem Cell Deficiency

Iatrogenic LSCD represents a significant complication associated with conjunctival tumor surgery, particularly in cases involving extensive limbal excision or long-term topical medication use. The limbal stem cells play a critical role in maintaining corneal epithelial homeostasis and facilitating tissue regeneration. Iatrogenic damage can compromise these functions, leading to progressive ocular surface pathology [126]. LSCD is characterized by impaired epithelial healing, chronic ocular surface inflammation, stromal melting, corneal conjunctivalization, and neovascularization. Clinical sequelae include PEDs, corneal scarring, and significant visual impairment, with superior quadrants often disproportionately affected due to surgical trauma at limbal sites. Contributing factors such as concurrent external ocular diseases (e.g., pterygium, keratoconjunctivitis sicca, and herpes simplex keratitis) and long-term exposure to toxic topical medications exacerbate the condition [127]. Surgical planning for conjunctival tumors should focus on minimizing limbal trauma, and in cases of ocular surface squamous neoplasia involving a substantial portion of the limbus, concomitant limbal stem cell transplantation should be considered for better long-term outcomes [128,129]. Lubrication, autologous serum eye drops, and amniotic membrane transplantation may also help manage epithelial defects.

##### Corneal Scarring

**Figure 12:**
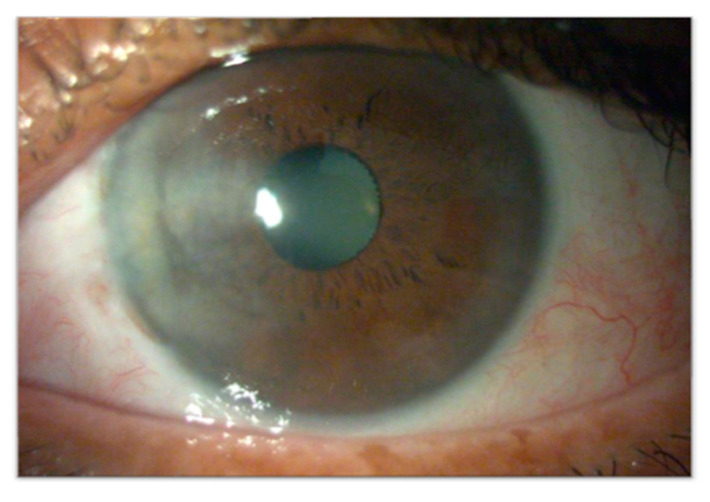
Iatrogenic corneal leucoma in the left eye of a 43-year-old patient after conjunctival intraepithelial neoplasia excision.

Corneal scarring represents a significant iatrogenic complication following conjunctival tumor surgery, particularly where the neoplastic involvement reaches the Bowman’s membrane (Figure 12).

This is often complicated further by the use of adjuvant therapies, such as cryotherapy or topical chemotherapy, which can exacerbate corneal damage and corneal inflammation. Scarring disrupts corneal transparency by causing irregular extracellular matrix deposition, stromal opacification, and vascularization, which collectively impair vision. Pro-inflammatory cytokines, such as interleukin-1 (IL-1) and tumor necrosis factor-alpha (TNF-α), promote fibroblast activation and extracellular matrix remodeling, leading to opacity in the stromal layer [130]. Strategies to prevent or minimize corneal scarring include “no-touch” surgical techniques and precise excision margins to reduce unnecessary stromal trauma. Additionally, adjuvant therapies such as amniotic membrane transplantation and autologous serum eye drops have been shown to reduce inflammation and enhance epithelial healing, thereby mitigating the risk of scarring [130]. Novel approaches, including growth factor-enriched formulations and matrix regenerating agents, are emerging as promising treatments to promote corneal transparency and prevent fibrosis [130,131,132].

##### Conjunctival Scarring and Symblepharon

**Figure 13:**
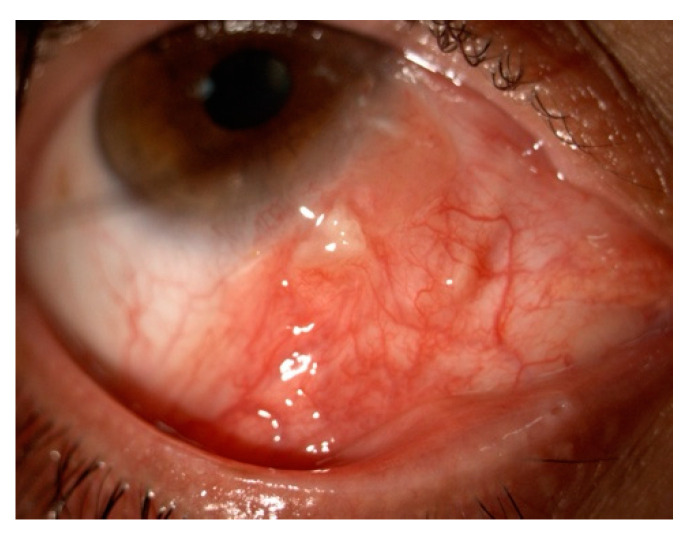
Postoperative conjunctival scarring following excision of an infero-nasal conjunctival tumor of the right eye in a 56-year-old patient.

Symblepharon is a relevant iatrogenic complication of conjunctival tumor surgery, often associated with extensive tissue manipulation or cicatricial changes (Figure 13).

**Figure 14:**
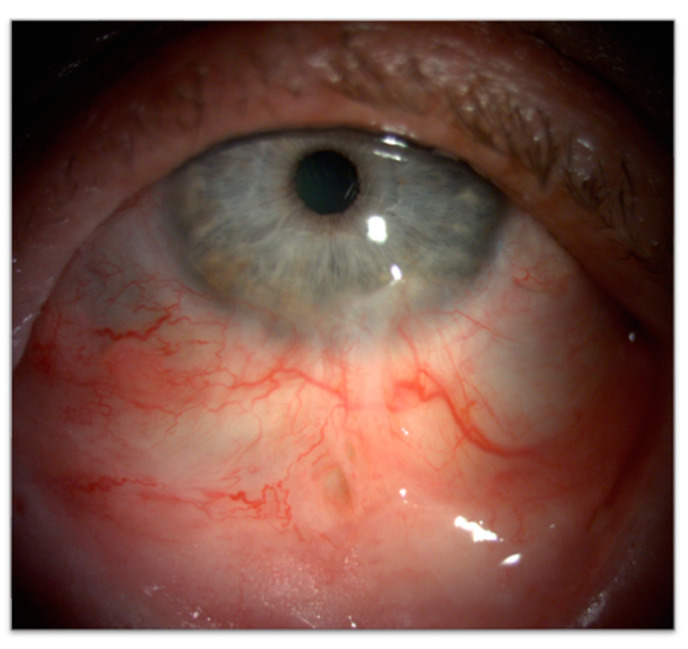
Postoperative symblepharon and fornix adhesion following extensive excision of a conjunctival tumor involving the tarsal conjunctiva in a 75-year-old patient.

This condition can lead to significant anatomical and functional disruptions, including fornix shortening, lagophthalmos, cicatricial ectropion, and exposure keratopathy which collectively exacerbate ocular surface disease and compromise visual rehabilitation [133]. Symblepharon pathogenesis is frequently linked to postoperative inflammation and fibrosis, where the healing process promotes adhesion formation. Early prevention is crucial to preserve fornix anatomy and limit the risk of adhesions (Figure 14).

The use of a symblepharon ring, typically made from polymethyl methacrylate and customizable to patient anatomy, is an effective intervention. These rings separate the tarsal and bulbar conjunctiva during the critical early postoperative period, preventing adhesions and promoting tissue healing [134]. For established or recurrent symblepharon, surgical management is often required. Techniques include meticulous symblepharolysis followed by reconstruction with tissue grafts such as amniotic membrane, autologous conjunctival, or oral mucosal grafts. Adjuvant therapies such as mitomycin C or subconjunctival corticosteroids are frequently employed to minimize recurrence. In refractory or severe cases, keratolimbal allografts have emerged as a promising approach, providing both a mechanical barrier against scar extension and a source of conjunctival epithelial stem cells to support ocular surface regeneration [133,135].

##### Dry Eye Disease After Conjunctival Tumor Management

As previously discussed, iatrogenic DED after conjunctival surgery is linked to conjunctival scarring, symblepharon, corneal scarring, limbal stem cell deficiency, persistent epithelial defects, and conjunctival inflammation. The pathogenesis involves three distinct mechanisms: reduced mucin secretion disrupts tear film adherence, aqueous deficiency reduces tear volume, and lipid layer dysfunction accelerates evaporative tear loss. Ocular surface inflammation perpetuates epithelial damage and neurotrophic dysfunction, further exacerbating the vicious cycle of DED [26,127,133,136]. Management approaches align with previously mentioned strategies, emphasizing the need to minimize surgical trauma and promote postoperative healing. These include preservative-free artificial tears; punctal occlusion; anti-inflammatory agents such as corticosteroids or cyclosporine; and scleral lenses and amniotic membrane transplantation for severe cases [137].

A comprehensive overview of the most frequent iatrogenic ocular surface complications following conjunctival tumor surgery is summarized in Table 3.

### 3.3. Tumors of the Lacrimal Gland

Lacrimal gland neoplasms constitute 10% of orbital space-occupying lesions, with an incidence of approximately 1 per million annually [138]. Patients typically present with a painless, progressively enlarging mass in the lacrimal gland area, which may be accompanied by proptosis, diplopia, or vision changes [139]. Diagnosis is primarily established through imaging studies, such as magnetic resonance imaging or computed tomography scans, and confirmed through histopathological examination [139]. Management often involves eye-sparing surgical resection of the tumor, followed by adjuvant radiotherapy or concurrent chemoradiation therapy to address residual disease and reduce recurrence risk [138]. Recent studies have shown that eye-sparing approaches, combined with adjuvant therapies, can achieve reasonable local control and survival rates while preserving ocular function [140].

#### Iatrogenic Ocular Surface Complication Following Lacrimal Gland Carcinoma Surgery

Surgical removal of the main lacrimal gland significantly reduces basal and reflex tear production, leading to a marked decrease in tear secretion immediately after surgery. However, studies demonstrate that accessory lacrimal glands can adequately compensate for the loss of the main lacrimal gland in most cases, maintaining a stable tear film and preventing long-term ocular surface damage. Furthermore, histological and protein analyses revealed similar tear protein compositions between the main and accessory glands [141]. Lacrimal gland dysfunction following surgery or radiation therapy not only impairs aqueous tear production but may also disrupt its neural and hormonal regulation, contributing to persistent ocular surface inflammation and dry eye disease [142]. As a matter of fact, patients with lacrimal gland carcinoma undergoing eye-sparing surgery followed by adjuvant radiation therapy face notable ocular complications [143]. A study by Zhao et al. reported DED as the most frequent complication, affecting 91% of the cases, followed by radiation retinopathy (70%) and cataract progression (48%). DED was primarily managed using artificial tears, punctal plugs, and scleral lenses. While 20 out of 21 patients with DED achieved symptomatic control, one patient developed severe keratitis that progressed to a refractory corneal ulcer, ultimately leading to corneal perforation, irreversible ocular damage, and the need for enucleation. The findings highlight the importance of careful radiation planning to minimize retinal and corneal exposure and mitigate complications. Despite these challenges, 57% of the patients achieved a final visual acuity of 20/40 or better [144].

Table 4 outlines the major ocular surface complications observed after lacrimal gland tumor resection and adjuvant radiotherapy.

### 3.4. Choroidal Tumors

Uveal melanoma (UM) is the most frequent primary intraocular malignancy in adults, accounting for 5% of all melanomas. Most cases of UMs consist of choroidal melanoma, while the remaining lesions arise from the ciliary body and the iris [145,146].

Standard treatments include high-dose brachytherapy and proton therapy (PT). In selected cases, external beam radiation therapy (EBRT) allows for the precise targeting of the tumor minimizing damage to surrounding tissues. Brachytherapy and PT often require surgical preparation: tantalum clips are placed on the sclera to delineate tumor margins, ensuring precise radiation delivery. While effective, brachytherapy and PT can result in ocular complications, including telangiectasia, conjunctival adhesions, damage to the lacrimal gland, meibomian glands, or conjunctival goblet cells. In addition, discomfort caused by visible or superficial clips may arise [147,148]. Alternative approaches, such as transpupillary thermotherapy and cryotherapy, have been explored for smaller or less aggressive melanomas but generally exhibit limited efficacy in achieving durable local control [149]. Nowadays, enucleation is generally reserved for cases where conservative treatments fail or for very large ocular melanomas [150]. The management of UMs located in the superotemporal region is particularly challenging because proximity to the lacrimal gland increases the risk of severe radiation-induced DED [151].

Brachytherapy, particularly with palladium-103 plaques, has become the preferred option for UM. This procedure involves the temporary suturing of radioactive plaques to the sclera overlying the tumor. Palladium-103 offers advantages over iodine-125, as its photons are absorbed more efficiently in the tissue, reducing irradiation to sensitive structures such as the fovea, the optic disk, and the lens. The plaques remain in place for 5 to 7 days before surgical removal [152]. Thanks to its localized radiation delivery, this technique is associated with fewer anterior segment complications, such as cataracts, neovascular glaucoma, and keratitis, compared to other radiation modalities [150].

In contrast, PT uses high-energy proton beams to deliver radiation, which is especially advantageous for treating UMs regardless of their size, shape, or location [147]. While effective for large and irregularly shaped tumors, PT carries a higher risk of collateral damage, particularly to the ocular surface and adnexa. The proximity of critical structures, such as the eyelid and lacrimal gland, poses challenges. Hypofractionated PT has been linked to lacrimal gland damage and an increased risk of DED [153]. To mitigate these risks, optimizing PT parameters by adjusting gaze direction and employing advanced shielding techniques has become essential for minimizing irradiation to adjacent tissues while ensuring effective tumor control [154].

#### 3.4.1. Iatrogenic Ocular Surface Complications After Uveal Melanoma Surgery

##### Tantalum Clips Exposure

**Figure 15:**
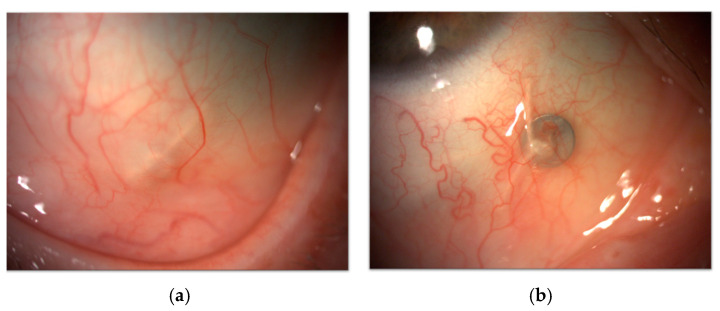
(**a**) Slit-lamp image showing a well-positioned episcleral brachytherapy clip for choroidal melanoma, partially visible beneath an intact conjunctival layer. (**b**) Example of an exposed episcleral clip due to conjunctival thinning, leading to ocular discomfort and localized conjunctival inflammation.

Surgical preparation is integral to the management of uveal melanoma, particularly for brachytherapy and PT. Both techniques rely on precise anatomical guidance to maximize treatment efficacy while minimizing damage to surrounding tissues. For PT, tantalum clips are strategically placed on the sclera to delineate tumor margins, enabling the precise delivery of proton beams. Similarly, brachytherapy involves the temporary suturing of radioactive plaques directly over the tumor site [152,154]. One significant iatrogenic complication is the visibility or superficial migration of tantalum clips. When these clips are inadequately covered by conjunctiva or in case of postoperative shift, they may cause persistent irritation, foreign body sensation, and ocular discomfort (Figure 15) [155].

Peripheral melanomas are particularly prone to this issue, as clips in these locations are more likely to become exposed. In such cases, the surgical removal of the clips may be necessary to alleviate symptoms and restore ocular comfort.

##### Conjunctival Scarring and Adhesions

Conjunctival scarring and adhesions arise from surgical manipulation for plaque insertion and radiation therapy. The surgical placement of tantalum clips disrupts conjunctival surface homeostasis, leading to adhesions between the conjunctiva and underlying scleral tissue. Furthermore, superficial migration of the clips can contribute to conjunctival scarring or adhesions, further compromising ocular surface integrity and motility. Scarring can also result in the formation of adhesions between the conjunctiva and underlying scleral tissue. Subclinical inflammation triggered by radiation further impairs its regenerative capacity, leading to delayed wound healing, PED, and fibrotic tissue deposition, thus exacerbating adhesion formation. Moreover, collateral damage to the lacrimal and meibomian glands, particularly in PT, aggravates DED and destabilizes the ocular surface [1,147]. To minimize such outcomes, meticulous surgical planning is crucial, with careful clip placement and proper conjunctival coverage to reduce trauma. Postoperative care, including regular monitoring, lubricants, or anti-inflammatory treatments, is essential to support conjunctival healing, prevent epithelial defects, and manage long-term ocular surface complications.

##### Dry Eye Disease After Uveal Melanoma Management

Several studies have demonstrated that radiation therapy for uveal melanoma frequently induces DED [156]. Factors such as tumor size, location, distance from the optic disk, and the percentage of retina irradiated have been identified as critical risk determinants. Eyelid rim irradiation, indirectly indicated by alopecia, exacerbates DED by damaging the meibomian, Zeiss, and Moll glands, leading to lipid tear deficiency and rapid aqueous evaporation [157]. Also, the aqueous component of the tear may be involved due to the irradiation of Krause accessory lacrimal glands located in the conjunctival fornices and Wolfring accessory lacrimal glands at the level of the non-marginal borders of the tarsal plate. The corneal epithelium and limbal stem cells are also affected, leading to progressive epithelial erosions, conjunctivalization, and scarring. Additionally, mechanical alterations of the tarsal surface can aggravate ocular surface irritation. The conjunctiva and the cornea are also vulnerable to radiation-induced damage [155]. Loss of conjunctival goblet cells reduces the mucin tear component, while irradiation of the limbus and corneal stem cells contributes to severe DED and LSCD. Long ciliary nerve involvement in cases of temporal melanoma further complicates outcomes, potentially leading to neurotrophic keratitis and corneal ulceration [158]. Efforts to optimize radiation delivery, such as anterior segment sparing and lid sparing, aim to mitigate these effects and preserve ocular function [148].

##### Lacrimal Gland Irradiation

Radiation therapy of the lacrimal gland frequently induces DED, with hypofractionated PT being a significant contributor [147]. Particularly, irradiation in superotemporal and temporal locations poses a significant risk. Lacrimal gland histopathological changes, such as serous acinar damage, atrophy, and necrosis, can manifest as early as 48 h after the initial dose of radiation [159]. A study involving 853 patients reported a five-year incidence of dry eye disease (DED) of 23.0%, with severe forms occurring in 10.9% of the patients. Superotemporal melanomas presented the highest risk for severe DED (hazard ratio [HR] 5.82), followed by temporal tumors (HR 2.63), with additional risk factors including age ≥70 years, ≥35% retina receiving ≥12 Gy, and eyelid rim irradiation. Although severe DED correlated with greater visual acuity deterioration, it did not significantly impact enucleation rates. The study concluded that while tumor location influences the risk of DED, it should not contraindicate PT, as visual outcomes and enucleation rates remain unaffected.

Table 5 provides an overview of ocular surface complications induced by brachytherapy and proton therapy for uveal melanoma.

### 3.5. Future Perspectives and Preventive Strategies

The management of iatrogenic ocular surface disease remains a critical aspect in the care of patients undergoing surgical or adjunctive treatments for ocular and adnexal tumors. Contemporary clinical strategies should emphasize risk stratification and preventive measures to reduce both the incidence and severity of these complications. Findings from tear film osmolarity, meibography, and monitoring of blink patterns may assist in stratifying patients by risk and informing tailored surgical and postoperative plans. Also, the implementation of advanced surgical techniques that address both inflammatory and regenerative approaches is essential to minimize collateral tissue damage. Finally, the integration of multidisciplinary care involving oculoplastic surgeons, oncologists, and ocular surface specialists is fundamental to achieving an optimal balance between oncologic efficacy and long-term functional rehabilitation.

## 4. Conclusions

Iatrogenic ocular surface disease following surgery for ocular and adnexal tumors presents a diverse spectrum of clinical manifestations that require tailored therapeutic approaches. These complications differ according to tumor localization and treatment and an early diagnosis is crucial in order to mitigate the risk of irreversible, sight-threatening complications. Eyelid tumor surgery often results in lower eyelid malposition, disrupting ocular surface integrity. Entropion may compromise ocular surface integrity by corneal irritation and epithelial defects. On the other hand, malpositions such as cicatricial ectropion, lagophthalmos, and lower eyelid retraction can result in exposure keratopathy, conjunctival inflammation, chronic dry eye disease, and impaired lacrimal drainage, leading to epiphora or insufficient tear clearance. These complications compromise ocular surface protection, increasing the risk of persistent epithelial defects, corneal scarring, and infections. Conjunctival tumor surgery may lead to extensive conjunctival scarring, symblepharon, persistent epithelial defects, and limbal stem cell deficiency. Surgical treatment of lacrimal gland tumors frequently reduces tear production, predisposing patients to chronic dry eye disease and an increased risk of keratitis, which, in severe cases, may progress to corneal ulceration, perforation, and irreversible stromal damage. The management of uveal melanoma often involves brachytherapy or proton therapy, where radiation exposure can compromise the ocular surface, resulting in conjunctival adhesions, tantalum clip exposure, dry eye disease, and, in some cases, lacrimal gland irradiation.

## Figures and Tables

**Table 1:** Search strategy and keywords regarding “eye”, “surgical approach”, “disease”, and “ocular complication”.

Eye		Surgical Approach		Disease		Ocular Complication
“eye” OR “ophthalmology” OR “ocular” OR “periocular” OR “orbit*” OR “eyelid*” OR “adnexal” OR “lacrimal” OR “ocular surface” OR “conjunctiv*” OR “choroid*”	AND	“blepharoplasty” OR “excision” OR “Mohs micrographic” OR “radiotherapy*” OR “chemotherap*“ OR “cryotherapy” OR “biopsy” OR “flap*” OR “graft*” OR “canthoplasty” OR “tarsal strip” OR “evisceratio*” OR “exenteratio” OR “proton” OR “brachytherapy”	AND	“carcinoma*” OR “melanoma*” OR “tumor*” OR “neoplas*” OR “lympho*”	AND	“eyelid malposition” OR “ectropion” OR “entropion” OR “lagophthalmos” OR “scleral show” OR “round eye” OR “symblepharon” OR “adhesion” OR “conjunctivitis” OR “epiphora” OR “keratopathy” OR “keratitis” OR “limbal stem cell” OR “epithelial defect” OR “epitheliopathy” OR “corneal scarring” OR “corneal ulcer*” OR “corneal perforation” OR “dry eye”

**Table 2:** Overview of iatrogenic ocular surface complications following eyelid tumor surgery, including main complications, pathophysiologic mechanisms, and management strategies. (DED, dry eye disease; LTS, lateral tarsal strip).

Iatrogenic Ocular Surface Complication Following Eyelid Tumor Surgery	Pathophysiologic Mechanisms	Management Strategies
Postoperative inflammatory response of the ocular surface	Immune-mediated inflammation; fibroblast activation; cytokine and growth factor release; and radiation and chemotherapy-induced cellular stress	Lubrication; anti-inflammatory agents (e.g., cyclosporine, lifitegrast); corticosteroids; serum eye drops; amniotic membrane; and autologous fat grafting
Lagophthalmos	Transient: postoperative edema, temporary facial nerve paresis, and local anesthesia Permanent: cutaneous scarring, excessive tissue removal, radiation-induced fibrosis, and nerve damage	Transient: eyelid taping, ointments, and temporary tarsorrhaphy Permanent: surgical revision, gold/platinum weights, skin grafts, tarsal strip procedures, and temporalis muscle transfer
Upper eyelid malposition	Mechanically altered eyelid-globe congruity; orbicularis oculi damage; anterior lamellar deficiency; and dynamic eyelid abnormalities	Surgical reconstruction (e.g., levator muscle elongation; levator aponeurosis reinsertion, Müller’s muscle myectomy, etc.); gold weight insertion; and autologous fat grafting
Lower eyelid malposition	Ectropion: vertical traction, anterior lamella shortening, gravitational forces, and cutaneous scarring Entropion: posterior lamella shortening, orbicularis muscle disinsertion, and cicatricial changes Round eye: incomplete globe coverage with scleral show	Ectropion: surgical reconstruction (e.g., LTS, canthoplasty, skin grafts, Reidy–Adamson flap, lipostructure, etc.), and steroid injections Entropion: surgical reconstruction (e.g., tarsal fracture, posterior lamellar grafts, anterior lamellar repositioning, etc.) Round eye: supportive lubrication and surgical reconstruction
Epiphora	Punctal displacement from medial ectropion or lower eyelid malposition; lacrimal pump failure; conjunctival adhesions; and post-surgical tear drainage obstruction	Eyelid repositioning; punctoplasty; conjunctivoplasty; LTS with conjunctival anchoring; punctal stenting; and scar revision
Exposure keratopathy, corneal ulcers, and infections	Tear film instability; corneal epithelial defects; microbial keratitis; altered blink dynamics; and mechanical trauma from eyelid malposition	Lubrication; punctal plugs; anti-inflammatory agents; antibiotics; eyelid taping; bandage contact lenses; and amniotic membrane
Dry eye disease (DED)	Reduced meibum secretion due to orbicularis and gland dysfunction; decreased blink efficacy; eyelid-globe incongruity; and lacrimal and goblet cell dysfunction	Lubrication; topical anti-inflammatory agents; punctal plugs; autologous serum; glandular function evaluation; and blink training

**Table 3:** Summary of iatrogenic ocular surface complications following conjunctival tumor surgery. Each complication is detailed with respect to its underlying pathophysiological mechanisms and recommended management strategies. (DED, dry eye disease; PED, persistent epithelial defect; LSCD, limbal stem cell deficiency; NGF, nerve growth factor; CD4+ T, Cluster of Differentiation 4 Positive T Lymphocytes; HLA-DR, Human Leukocyte Antigen—DR isotype; ICAM-1, intercellular adhesion molecule-1).

**Main Ocular Surface Complications**	**Pathophysiologic Mechanisms**	**Management Strategies**
Conjunctival inflammation	Immune-mediated inflammatory cascade; CD4+ T-cell infiltration; overexpression of HLA-DR and ICAM-1; and epithelial instability triggered by surgical trauma and adjuvant therapies	Topical anti-inflammatory agents (corticosteroids, cyclosporine, etc.); lubrication; and early anti-inflammatory modulation
PED	Corneal epithelial barrier disruption; limbal damage; cryotherapy-induced toxicity; delayed wound healing; and excessive matrix metalloproteinase activity	Preservative-free artificial tears; punctal occlusion; bandage contact lens; autologous serum eye drops; amniotic membrane transplantation; scleral lenses; and topical growth factors (e.g., NGF, etc.)
LSCD	Iatrogenic limbal trauma; stem cell depletion; chronic inflammation; corneal conjunctivalization; and exposure to topical chemotherapy	Limbal stem cell transplantation; amniotic membrane transplantation; intensive lubrication; topical cyclosporine; and autologous serum drops
Corneal scarring	Involvement of Bowman’s layer; excessive fibroblast activation; cytokine-mediated stromal remodeling (e.g., IL-1, TNF-α); and radiation or cryotherapy-induced damage	Prevention via “no-touch” technique; amniotic membrane; autologous serum; anti-fibrotic therapies; and matrix-regenerating agents
Conjunctival scarring and symblepharon	Extensive conjunctival manipulation; postoperative fibrosis; adhesions between tarsal and bulbar conjunctiva; and chronic inflammation and delayed healing	Symblepharon ring for prophylaxis; surgical symblepharolysis; reconstruction with conjunctival, oral mucosal, or amniotic membrane grafts; adjunctive mitomycin C or corticosteroids; and keratolimbal allograft in refractory cases
DED	Reduced mucin (goblet cell loss), aqueous (lacrimal injury), and lipid (meibomian gland dysfunction) components; tear film instability; chronic inflammation; corneal nerve injury; and goblet cell toxicity due to topical chemotherapy	Lubrication with monitoring and modulation of tear film components (mucin, lipid, aqueous); anti-inflammatory therapy (cyclosporine, corticosteroids, etc.); punctal plugs; scleral lenses; amniotic membrane; and epithelial neurotrophic support (e.g., NGF)

**Table 4:** Summary of iatrogenic ocular surface complications following lacrimal gland tumor treatment (DED, dry eye disease; PED, persistent epithelial defect).

Main Ocular Surface Complications after Lacrimal Gland Carcinoma Surgery	Pathophysiologic Mechanisms	Management Strategies
Severe aqueous-deficient DED, keratitis, neurotrophic keratitis, PED, corneal ulcer, in refractory cases corneal perforation	Loss of lacrimal gland function, radiation-induced glandular atrophy and neural dysfunction, and inflammatory milieu	Lubrication, punctal plugs, scleral lenses, and topical anti-inflammatory agents (e.g., cyclosporine, etc.)

**Table 5:** Summary of iatrogenic ocular surface complications following uveal melanoma treatment (DED, dry eye disease; LSCD, limbal stem cell deficiency; PT, proton therapy).

Main Ocular Surface Complications After Uveal Melanoma Surgery	Pathophysiologic Mechanisms	Management Strategies
Tantalum clip exposure	Mechanical conjunctival trauma from clip placement, superficial migration, and radiation-induced conjunctival thinning	Surgical clip removal if exposed, topical lubrication, conjunctival repair, and shielding during PT
Conjunctival scarring and adhesions	Surgical dissection trauma, subclinical inflammation, and fibrosis triggered by radiation and surgical manipulation	Lubrication, anti-inflammatory therapy (e.g., topical corticosteroids), amniotic membrane grafts, and conjunctivoplasty if needed
LSCD	Radiation injury to the limbus, ischemic insult, progressive stem cell loss, and epithelial instability	Limbal stem cell transplantation, amniotic membrane transplantation, intensive lubrication, and topical steroids or growth factors
Lacrimal gland dysfunction and irradiation	Direct radiation damage to main and accessory lacrimal glands, inflammatory and fibrotic changes, and neural deregulation	Lubrication, punctal occlusion, scleral contact lenses, and shielding during PT
DED	Goblet cell loss, meibomian gland dysfunction, radiation-induced lacrimal, and accessory gland damage	Lubrication, punctal plugs, topical cyclosporine/lifitegrast, scleral lenses, autologous serum, and anti-inflammatory therapy

## Abbreviations

The following abbreviations are used in this manuscript:

BCC	basal cell carcinoma
CD4+ T cell	Cluster of Differentiation 4-positive T lymphocyte
CIN	conjunctival intraepithelial neoplasia
CoM	conjunctival melanoma
DED	dry eye disease
EBRT	external beam radiation therapy
EMZL	extranodal marginal zone B-cell lymphoma
HR	hazard ratio
HLA-DR	Human Leukocyte Antigen—DR isotype
ICAM-1	intercellular adhesion molecule 1
IFNα2b	interferon alpha-2b
IL-1	interleukin-1
LSCD	limbal stem cell deficiency
LTS	lateral tarsal strip
MALT	Mucosa-Associated Lymphoid Tissue
MCC	Merkel cell carcinoma
MMC	mitomycin C
NGF	nerve growth factor
NHL	Non-Hodgkin Lymphoma
OAL	ocular adnexal lymphoma
OSSN	ocular surface squamous neoplasia
PED	persistent epithelial defect
PT	proton therapy
SCC	squamous cell carcinoma
UM	uveal melanoma
